# Association between grades of Hydronephrosis and detection of urinary stones by ultrasound imaging

**DOI:** 10.12669/pjms.344.14602

**Published:** 2018

**Authors:** Sultan Abdulwadoud Alshoabi

**Affiliations:** 1 Dr. Sultan Abdulwadoud Alshoabi, MBBS, MD. Arab board and Jordanian Board of Radiology, Assistant Professor of Radiology, College of Applied Medical Sciences, Taibah University, Almadinah Almunawwarah, Saudi Arabia

**Keywords:** Grades, Hydronephrosis, Stone detection, Ultrasound imaging

## Abstract

**Objective:**

To correlate between hydronephrosis grades and detection of urinary stones by B-mode ultrasound imaging.

**Methods:**

This study included 210 ultrasound reports of patients who underwent abdominal ultrasound imaging in the period from 1st January 2016 to 31st October 2017, and diagnosed as hydronephrosis. Data collected from the ultrasound reports. The detection rates of stones using B-mode ultrasound imaging compared in different grades of hydronephrosis. Chi-square test and Odds Ratio (OR) were performed to assess the relationship between variables.

**Results:**

Of 210 patients, hydronephrosis was unilateral in 91.8% of patients and bilateral in 8.1%. It was distributed in grade 2, grade 3, grade 1 and grade 4 in 58.57%, 20%, 12.38% and 9.1% of the patients respectively. B-mode ultrasound imaging determined the cause of hydronephrosis in 65.2% of cases. Urinary stones were the cause in 60% of the patients. The detection rate of urinary stones was 50%, 61% and 71.4% for grades 1, 2 and 3 hydronephroses respectively. On simple logistic regression analysis, urinary stones detected in Grade-3 were four times more compared to that in grade 4 (P=0.016) (OR 4.125, 95% CI 1.29-13.136%).

**Conclusion:**

Detection of urinary stones as the cause of hydronephrosis increases with increasing the grade of hydronephrosis from Grade-I to Grade-III and decrease in Grade-IV. Urinary stones were the cause of hydronephrosis in 60% of the patients in this study.

## INTRODUCTION

Hydronephrosis is dilatation of the pelvicalyceal system as a result of urine excretion failure. It is either due to obstruction or non-obstructive causes. It is a common medical problem encountered by the primary healthcare workers, emergency physicians, and urologist’s worldwide.^1^ Hydronephrosis divided into obstructive and non-obstructive. Obstructive usually caused by urinary stones, blood clots, ureteropelvic junction (UPJ) obstruction, stricture or external compression by tumor, fibrosis or others. The blockage may be acute or chronic, unilateral or bilateral, partial or complete. Non-obstructive caused by reflux, residual or others.^1^,^2^

The Society of Fetal Urology (SFU) grading system classified hydronephrosis into five grades. Grade 0 = no hydronephrosis. Grade 1 = dilatation of renal pelvis only. Grade 2 = grade 1 + dilatation of few calyces. Grade 3 = grade 2 + dilatation of all calyces. Grade 4 = grade 3 + thinning of the renal parenchyma.^3^

Ultrasound Imaging is a noninvasive, non-expensive, widely available imaging modality. It can achieve accurate diagnosis in the most cases of acute and chronic renal obstruction without the need for radiation.^4^ However, it is a widely accepted, a very useful and reasonably accurate to diagnose hydronephrosis; it is not very effective to determine the cause of obstruction.^5^ Ultrasound imaging can easily detect kidney stones. It can identify ureteric stones with an efficacy up to 57.3% and 81.3% without and with hydronephrosis respectively.^6^ Ultrasound imaging is less sensitive than CT for detecting of ureteric stones, but it recommended as the first choice imaging modality especially in pregnant and pediatric patients.^7^

This study aimed to determine the effect of grades of hydronephrosis on the detection rate of urinary stones as the most common cause. This is a very common clinical problem in emergency room, outpatient clinics and even in private clinics. Detection of location of urinary stone and its size is a critical point for planning of the treatment. Ultrasound imaging is selected for this study as it is commonly used, universally available and the first choice and radiation free.

This work will benefit ultrasonographers, radiologic technologists, radiologists, urologists and emergency practioners who are always interested in detecting the cause of hydronephrosis as frequent and important medical problem corresponds to their daily work.

## METHODS

This cross-sectional, retrospective study included 210 ultrasound reports of patients who had hydronephrosis (141 males and 69 females), at the ultrasound unit of the hospital. The patients underwent abdominal ultrasound imaging in the period from 1st January 2016 and 31st October 2017. The study included adults and children patients. Exclusion criteria were as follows:

1. Elderly patients.
2. Patients in whom bladder outlet obstruction was the cause of backpressure
3. Patients with apparent cause of hydronephrosis in the urinary bladder or distal,
4. Infant patients in whom congenital anomalies usually the cause. Data collected from the patient’s ultrasound reports. The study was approved by the Hospital Ethics Committee.

An ultrasound machine Medison, Sono ex-model 6 was used. All patients scanned in the supine position by the same Radiologist. Ultrasound scanning was done in a slightly dark room to minimize the reflected artifact of the screen. A curved transducer of 3.5 MHz was used.

Data entered and analyzed by using the Statistical Package for the Social Sciences (SPSS), version 16. Descriptive statistics used frequencies and percentages. Chi-square test and simple logistic regression performed. Statistical significant difference assumed for P-value ≤0.05

## RESULTS

This study included 210 ultrasound reports of hydronephrosis patients. Males account for approximately 67.14% of the sample (67.14% vs. 32.86%). The majority of patients (91.8%) revealed unilateral hydronephrosis. The right kidney affected more than the left (52.7% vs. 41.4%). The sample revealed grade 2, grade 3, grade 1 and grade 4 hydronephrosis in 58.57%, 20%, 12.38% and 9.1% of the patients respectively (Table-I). B-mode ultrasound imaging detected the cause of hydronephrosis in 65.2% of all patients. Urinary stones were the cause in 60% of the whole patients (126 out of 210) The detection rate of stones increases with increasing the grade of hydronephrosis from grade-1 to grade-3 then drops in Grade-IV.

**Table-I:** Detected stones in different grades of hydronephrosis.

Grade	SFU grading system of Hydronephrosis (Now it is used in adults as well)	Detection rate of stones as the cause of hydronephrosis N (%)	Nondetermined +Other causes N (%)	Total N (%)
Grade 1	Dilated only renal pelvis	13 (50)	13 (50)	26 (12.38)
Grade 2	1 + dilated few calyces	75 (61)	48 (39)	123 (58.57)
Grade 3	2 + dilated all calyces	30 (71.4)	12 (28.6)	42 (20)
Grade 4	3 + thinning of the renal Parenchyma	8 (42.1)	11 (57.9)	19 (9.1)
		126 (60%)	84 (40%)	210 (100%)

The rate of the detection of urinary stones increases with increasing the grade of hydronephrosis from grade 1 up to grade 3 then drops in grade 4.

Urinary stones detected in Grade-III four times more than that in grade 4 (Odds ratio = 4.125, 95% confidence interval [Cl]=1.29-13.136%) and (P=0.016) (Table-II). The determined stones distributed in the ureterovesical junction (UVJ), renal pelvis, ureteropelvic junction (UPJ), upper ureter, lower ureter and midureter in 36.5%, 23%, 16.7%, 11.9%, 7.1% and 4.8% respectively (Table-III).

**Table-II:** Comparison between detection and the grades of hydronephrosis.

	P-value	Odds Ratio (OR)	95.0% C.I. for OR	
			Lower	Upper
Grade (1)	0.516	1.490	0.448	4.956
Grade (2)	0.090	2.344	0.876	6.269
Grade (3)	0.016	4.125	1.295	13.136

Simple logistic regression revealed significant relationship between detection rate of stones &the grades of hydronephrosis (Grade-4 was the reference of comparison).

**Table-III:** Site of the determined stones in hydronephrosis.

Site of the detected stone	Number of cases	% of cases
Ureteropelvic junction	21	16.7
Upper half of ureter	15	11.9
Middle part of ureter	6	4.8
Lower half of ureter	9	7.1
Ureteropelvic junction	46	36.5
Renal pelvis	29	23
Total	126	100

Table revealed significant relationship between the location of stone and its detection by ultrasound imaging (p=0.026).

**Fig.1:**
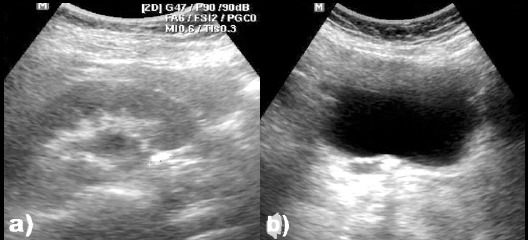
Ultrasound images for a) 10 mm stone in the right UPJ causing mild hydronephrosis, and b) Stone in the right UVJ causing hydroureteronephrosis.

## DISCUSSION

Extensive use of ultrasound imaging for diagnosis of renal problems has led to an increased rate of diagnosis of hydronephrosis in the last decades. In this study, we focused on the ability of ultrasound imaging to determine the urinary stones in the upper urinary tract in grades of hydronephrosis. This study found a significant relationship between detection of stone and the grade of hydronephrosis.

This study found that kidney and ureteric stones were the most common cause of hydronephrosis. These results are consistent with a previous study by Suzan et al. (2015), who reported that kidney and ureteric stones were the cause of hydronephrosis in 54% of adult patients.^8^ The gender distribution of the patients in the sample of this study was 67.14% males vs. 32.86% females. This result is compatible with previous studies by Hall (2009) and Nuraj P et al. (2017), who reported that renal calculi in males were nearly twice as it was in females.^9^,^10^

Distribution of the grades of hydronephrosis in this study is similar to a previous survey of Nuraj P et al. (2017), who reported that the grades of hydronephrosis were as follows: 48%grade 2, 22.8% grade 1, 16.2% grade 3 and 12.5% grade 4.^10^ In this study, there are significant associations between the stone length and location with the grade of hydronephrosis whereas the patient’s gender has no relationship with the grade. These results are consistent with a previous study by Song Y et al. (2015) regarding the stone length and patient’s gender but not compatible with him regarding the stone location.^11^ The rate of stone detection increased from grade 1 to 3 then drop in grade 4. These results are nearly compatible with a previous study by Ozden E et al. (2002) in grades 1, 2 and three but not consistent in grade 4.^12^ The drop in stone detection in grade 4 may be due to chronic causes other than a stone.

Unfortunately, we could not find any recent studies to compare with these results. These results can support a previous study by Goertz and Lotterman. (2010), who reported that increasing the grade of hydronephrosis had a predictive value to the size of the stone.^13^ In the current study, the least common location of the detected stones was the middle part of the ureter (4.8%). This result is compatible with the results of Hansen KL et al. (2016) who reported that ureteric stones are not easily detecting by ultrasound imaging due to bowel gases that usually obscure insonation window.^14^

In this study, we did not focus on the relationship of urinary stone size and the grade of hydronephrosis. In a previous study was done by Riddell et al (2014), the authors have reported that detection of hydronephrosis by ultrasound imaging increase with increasing the size of ureteric stone with a sensitivity of 75% in stones<6 mm upto 90% in stones >6 mm.^15^

### Recommendation

Further studies about the relationship between size of urinary stone and the grade of hydronephrosis by using CT that is more accurate imaging modality for measuring of the size of urinary stones are needed.

### Limitations of the study

1. This was a single-center study.
2. The ages of the patients were available only by categories (child, adult and elderly) and not by years.

## CONCLUSION

Detection rate of the urinary stones as the cause of hydronephrosis increases with increasing the grade of hydronephrosis from grade 1 to 3 and decrease in grade 4. Urinary stones were the cause of hydronephrosis in 60% of the patients in this study. The location of the stone is an important factor for its detection by ultrasound imaging. The ureterovesical junction was the most common site of the detected stones.
